# Spatio-temporal variability and pollution sources identification of the surface sediments of Shatt Al-Arab River, Southern Iraq

**DOI:** 10.1038/s41598-020-63893-w

**Published:** 2020-04-24

**Authors:** Hadi Allafta, Christian Opp

**Affiliations:** Faculty of Geography, Philipps-University of Marburg, Deutschhausstr. 10, 35037 Marburg, Germany

**Keywords:** Environmental sciences, Hydrology

## Abstract

Water draining from heavily industrialized basins introduces significant amounts of pollutants to the rivers water and sediments. Heavy industrial activities in the Shatt Al-Arab basin result in increased pollutant loads to the river’s surface sediments. Therefore, it becomes crucial to investigate the influence of anthropogenic activities on both spatial and temporal scales. This study unfolded the extent, sources, and distributions of heavy metals pollution in the sediments of the Shatt Al-Arab River. Extensive samplings were performed during the dry and the wet seasons at 25 stations along the river course for the analysis of 11 heavy metals. The analysis revealed high pollution levels in the river sediments compared to both their historical values and international standards. Statistical analysis techniques such as Principal Component Analysis (PCA) and Factor Analysis (FA) were applied. Statistical analysis showed that all the elements were well represented by four varifactors that explained a cumulative total variance of 74%. PCA/FA indicated that most investigated metals were of anthropogenic origins (i.e., industrial, residential, and agricultural sources). Pollution indices that were applied, such as Contamination Factor (CF) and Nemerow Pollution index (P_N_), indicated that sediments were: (i) considerably contaminated with Fe and Mo (ii) moderately contaminated with Cr, Zn, Ni, Cu, Pb and Mn and (iii) not contaminated with Co and V. The P_N_ values indicated serious pollution in the river sediments in all sites, even though the pollution was not evenly distributed, i.e., the upstream reaches of the river were more polluted compared to the downstream parts. In contrast to many studies that have reported changes in heavy metals concentrations due to seasonal variations, our data showed no significant relationship between metals concentrations and seasonality. This study addresses several of the major limitations of the current knowledge on this river’s pollution sources and analysis, such as the limited number of analyzed pollutants and restricted samplings in the current literature. The findings necessitate the implementation of effective management strategies to control pollution in the river basin.

## Introduction

Basra Governorate, the economic capital of Iraq, is the third-largest city in the country with a population of around three million^1^. Considered to be the center of the oil industry in Iraq, Basra contributes significantly to the growing national economy (above 70% of the gross national product)^2^. However, the economic growth that the country is experiencing has come at a high cost. Such growth has caused severe environmental degradations that not only threatens the environment but also made the resulting economic growth difficult to maintain^3,4^. The Shatt Al-Arab River, formed by the confluence of the Euphrates and Tigris rivers and empties in the Arabian Gulf, is the primary freshwater source in a rather arid area surrounding Basra^5^. The river provides lifeline benefits shared by millions of people living within its basin^6^. Water needed to sustain domestic, agricultural, industrial, natural ecosystems, transportation, and recreational purposes is mainly provided by the river^4^. Moreover, the river constitutes the main freshwater source for the Arabian Gulf and plays a crucial role in supporting the marine habitats in the north-eastern coastal areas of the Gulf^7,8^. However, the Shatt Al-Arab water quality has remarkably deteriorated in the last three decades due to anthropogenic activities. The increasing amounts of untreated wastewater and runoff that the river receives from the surrounding oil production fields^9^, urbanized areas^10^, and agricultural lands^11^ have resulted in a declined water quality in the river waterway^12,13^. Moreover, the Mesopotamian marshes draining into the Shatt Al-Arab that were once acting as a powerful filter for pollutants^14^ are still far from adequate restoration after the deliberate desiccation in the 1990s^15–17^. Therefore, the vital functions that the Shatt Al-Arab plays in sustaining healthy communities and maintaining a balanced ecosystem in the northern Gulf are greatly jeopardized^18^.

Owing to lack of proper wastewater treatment facilities in such a developing country, anthropogenic effluents are often directly discharged or flushed via runoff into the river^19^. Anthropogenic effluents are widely documented to increase heavy metals concentrations considerably in rivers^20,21^. After being discharged into rivers, such metals can adhere to particulate matter and eventually deposit in sediments^22–24^. Contaminated sediments can impose an adverse impact on benthic fauna^25,26^ and flora^27^. Sediment contaminants tend to accumulate in the biological tissues of some organisms, and when larger consumers (including human) feed on such organisms, the contaminants are then transferred into their bodies^17^. Moreover, the non-biodegradability of heavy metals is usually resulting in their accumulation in the environment^28^. Depending on their sorption properties, heavy metals can ultimately be partially or entirely released back into the water^29,30^, thereby threatening the aquatic life^31^. Thus, heavy metals contamination in sediments has negative implications on water quality, and their bioaccumulation in aquatic biota results in long-term impacts on human and ecosystem health^32^.

In recent years, however, concerns have emerged over the increasing prevalence of heavy metals in the Shatt Al-Arab River. Several research studies conducted on the assessment of pollution status in the river sediments have contributed to a better understanding of the river pollution in terms of sources, mechanisms, and management strategies^12,33–35^. These studies, however, were mostly constrained either to a specific reach within the river stretch or were limited to few and sparse stations along the river course. Therefore, these studies are not capable of comprehensively identifying the spatial distribution characteristics of the pollutants in the Shatt Al-Arab sediments. Furthermore, wastewater effluents from municipal and industrial activities in riparian zones represent a constant pollution source^36^. On the other hand, effluents from far uplands are mostly controlled by surface runoff and can be considered a seasonal phenomenon that is generally influenced by climate conditions within the river basin^36^. Most of the prior research has not covered the influence of seasonality as a key factor in controlling the sources of pollutants in the Shatt Al-Arab sediments. Understanding the trends of seasonal variations is critical to deriving adaptive strategies for effective basin management^37–39^ found that as the river flow decreases in the dry season, the rate of sedimentation and thus the metals concentrations increase. In the wet season, however, higher river flow produces a dilution effect, and thus, metals concentrations in sediment decline. Investigating the temporal variations of pollution in East River (Dongjiang) in China, ^40^ found that the dry season represents a crucial period for point source pollution due to the relatively lower river flow, whereas the end of the dry season and the beginning of the wet season is a critical time for nonpoint source pollution due to the agricultural return flows and the flushing effects of overland flows.

The primary step to controlling and treating heavy metal pollution is to investigate the pollution status, which often requires obtaining information from environmental monitoring data^41^. The multivariate statistical analysis techniques are effective tools in the interpretation of complex environmental data sets to: identify the possible pollution sources, understand the ecological status of the investigated systems, offer a valuable way for solid management of water resources, and provide rapid solution to pollution problems^42–44^. Multivariate statistical analysis methods (e.g., principal component analysis and factor analysis) have been used in the present study to characterize and evaluate surface sediment quality, and to identify the sources controlling metal pollution. Moreover, for adequate evaluation of pollution with heavy metals, pollution indicators can be used as guides for a detailed evaluation of the state of the sediment environment^45,46^. In the current study, these indicators were applied to examine whether the presence of heavy metals in the Shatt Al-Arab sediments was due to natural processes or anthropogenic activities.

Thus, the main objectives of the current study are: (i) to explore the spatial distribution of heavy metals in the Shatt Al-Arab surface sediments, (ii) to identify the origin of these elements using multivariate statistical analyses, (iii) to assess the levels of element contaminations in sediments using pollution indices, and (iv) to envisage the seasonal variations of metals concentrations in sediments during dry and wet seasons.

## Study site

The study area is situated within the Mesopotamia Plain of the Outer Platform^47^. The river watershed is mainly covered by Holocene alluvial sediments with marine influence in the southern parts at the Arabian Gulf coasts^48,49^. Originating from the confluence of the Euphrates and Tigris rivers at the Qurna City, the Shatt Al-Arab River flows to the southwest for 101 km before it constitutes the boundary between Iraq and Iran for the last 91 km of its main course until draining into the Arabian Gulf. The Shatt Al-Arab has a total length of 192 km, and a width ranging from 300 m at its origin to 700 m near the Basra City and around 800 m at its mouth^50^. The river has a depth that ranges between 8-15 m, considering tides^51^. The river watershed is generally characterized by flat, low-gradient landscapes of around 1 cm/km^52^. An area of approximately 145 km^2^ drains directly to the river basin downstream of the confluence of the Euphrates and Tigris rivers (excluding the Euphrates and Tigris Basin areas)^53^. The hydrological status of the river watershed is governed by conditions in the upper reaches of the feeding rivers, tides resulting from the seawater movement of the Arabian Gulf, and the effect of climatic conditions (i.e., precipitation, temperature, evapotranspiration, and runoff) on discharge rates and solids load in the river^54^. The Shatt Al-Arab watershed is characterized by a continental climate that ranges from sub-tropical, hot, and dry summer to cold and rainy winter. In summer, the average temperature in the shade is around 45 °C in the day time and drops to around 30 °C at night. In winter, temperature ranges between 18 and 2 °C during the day and night, respectively. The average annual precipitation in the region is about 100 mm^55^.

## Methods

### Sampling

**Figure 1:**
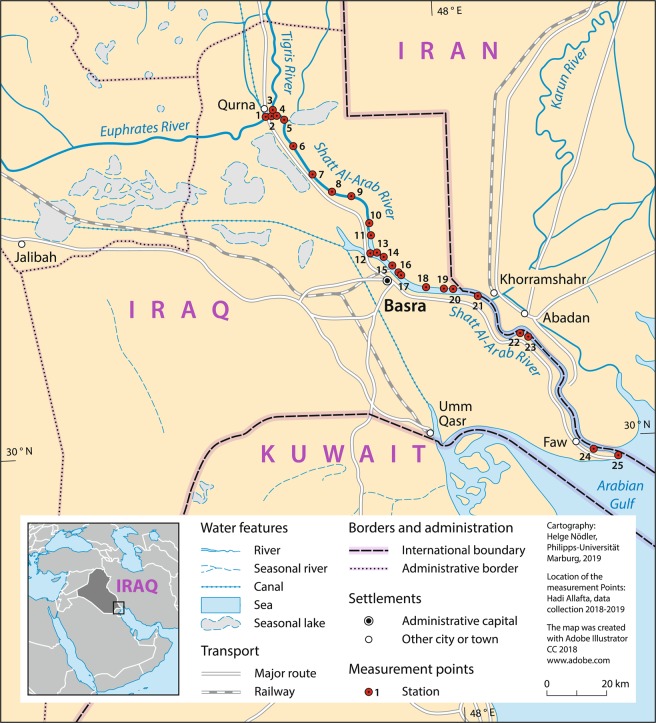
Study site.

Sediment samples were collected from 25 sampling stations on the 21^st^ of October 2018 (dry season) and the 21^st^ of January 2019 (wet season). The sampling scheme was designed to evaluate the seasonal variations in surface sediment contamination. The sampling locations were selected to capture the major transitions in the land cover/land use. Therefore, the monitoring locations were thoroughly representing the characteristics of the river basin, and they were not restricted to specific nodal information. One sediment sample from each station was obtained during each season. Geographical positions of sampling sites were measured with a portable GPS. Two sampling sites (sites 1 and 2) and one sampling site (site 3) lie on the Euphrates and Tigris rivers respectively before their confluence, and the other sites (i.e., 4–25) are along the entire Shatt Al-Arab River main course (Fig. 1). Water temperature (WT), pH, and total dissolved solids (TDS) were measured on-site using Hanna multi-parameter probe. We collected samples at a distance from the riverbanks to avoid possible contamination from the bank material^56^. The sampling was accomplished as per the United States Environmental Protection Agency USEPA (2014)^57^ by wading into the river while facing upstream (into the current), scooping the top 5 cm of the sediments in the upstream direction using a stainless steel scoop. We then carefully removed/drained excess water from the scoop to prevent/minimize the loss of fine-grained particles associated with the substrate being sampled. About one kilogram of sediment from each sampling site collected is then placed in a labelled glass pan and the cap is tightly secured. Thereafter, we shipped the samples to the laboratory and dried them at room temperature 27-35 °C for two weeks. The samples were then gently pulverized with an agate mortar and afterward were sieved with the standard sieve of 63 µm. It has been proposed to use the <63 µm fraction for heavy metals measurements^58^ due to many reasons: The heavy metals have been found to be available mainly in the clay/silt particles, this fraction is nearly equivalent to the materials carried in suspension, sieving does not alter heavy metal concentrations by remobilization, and numerous studies on heavy metals have been performed on the <63 µm fraction^59^. Seven grams of each sample were used to determine the element concentrations. Other researchers have utilized similar sampling and experimental specifications (e.g.^56^). The concentrations of heavy metals were measured using X-ray fluorescence (XRF) Spectrometer, SPECTRO XEPOS-2006 device at the Iraqi-German Laboratory at the Department of Geology, University of Baghdad.

Certainly, we admit that sampling just one time in each season can represent a source of uncertainty in our analyses. However, that was the optimum executable scheme currently in the study area, taking into account the security concerns in an unstable region, with almost half of the river course represents unsettled border between Iraq and Iran.

### Statistical analysis

Correlation matrix, which is a statistical method showing correlation coefficients between variables (14 variable in the current study), is expected to produce a large matrix (14 × 14), which is not the best technique to summarize the information in the current large data set. Alternatively, multivariate statistical methods, such as principal component analysis can substantially reduce the dimensionality of data containing a large set of variables^60^. This can be accomplished by converting the initial variables to a new small group of variables without missing the most important information in the initial data set. The new variable sets correspond to a linear combination of the initial data that are called principal components^61^. To perform a valid PCA, several prerequisites (e.g. Kolmogorov-Smirnov, Kaiser-Meyer-Olkin (KMO), and Bartlett’s tests) should be achieved first^62^. The Kolmogorov-Smirnov statistical test was applied to investigate the fitness of the data to log-normal distribution^63^. Kolmogorov-Smirnov test revealed that all the investigated variables had log-normal distribution with a confidence of 95% or higher. Furthermore, to test the data suitability for PCA, the Kaiser-Meyer-Olkin (KMO) test was performed. KMO test estimates sampling adequacy that expresses the proportion of variance among the investigated variables that could be a common variance. High KMO values indicate the usefulness of PCA, and some authors recommend a KMO value higher than 0.5^64–66^, which is the case in the present study where KMO equals 0.76. Moreover, Bartlett’s test of sphericity was performed to examine whether the correlation matrix is an identity matrix. The null hypothesis of Bartlett’s test is that the correlation matrix equals the unit matrix against the alternative hypothesis that the two matrices are unequal. In the current study, the null hypothesis is rejected as the significance level was 0 (less than 0.05), which implies that there are significant relationships among variables^67^.

While PCA reduces the contribution of variables of minor significance, factor analysis (FA), when follows PCA, can further reduce the contribution of variables of less significance resulting in even more simplification of the data structure obtained from PCA^68^. This goal can be accomplished by rotation of axes defined by PCA in accordance with well-established rules, and creating new variable sets called varifactors (VFs)^69^. Rotation of principal components results in a more straightforward and more expressive depiction of the underlying factors by reducing the contribution to principal components of less significant variables and enhancing the contribution to principal components of more significant variables. Principal components rotation generates a new factors set; each factor includes mainly a subgroup of the original variables with the least possible overlap so that the original variables are partitioned into new independent groups^70^. While the principal component is basically a linear combination of observable variables, the varifactor can encompass unobservable, latent, hypothetical variables^71^. Prior to performing PCA and FA, we standardized our data through z-scale transformation to prevent any misclassifications originating from the different orders of magnitude of numerical data values and variance of parameters^72,73^. PCA and FA were performed using (SPSS version 25) to determine the agglomeration of elements and eventually to identify the sources of elements in the river surface sediments^74,75^.

### Pollution analysis

The pollution indices were developed for evaluating soil and sediment quality^76^. Various authors^77–79^ have proposed the pollution impact ranges to convert the calculated numerical results into descriptive spectra of pollution that range from low to high intensity. Sediment quality guidelines provide values that permit quantification of sediment pollution and eventually make an overall assessment of the metal pollution degree in a river or marine sediments^80^. Pollution indices such as contamination factor (CF) are handy tools for the assessment of metal contamination in sediments and widely used for analyzing and transferring environmental information to decision-makers, managers, and the public^81^. The level of contamination for a metal in a particular area is expressed by the contamination factor (CF). It is the ratio of measured concentration and background concentration of a pollutant^77^, and calculated by the following formula:

1 $$CF={C}_{m{\rm{Sample}}}/{C}_{m{\rm{Background}}}$$

where *C*_*m* Sample_ is the concentration of a metal in sediment, and *C*_*m* Background_ is the background concentration of that metal in sediment^82,83^. As background concentrations for this area, ^33^ values were used as background values. ^77^ classified contamination factor values into four groups, i.e., CF < 1 represents low contamination, 1 ≤ CF < 3 indicates moderate contamination, 3 ≤ CF < 6 represents considerable contamination, and CF ≥ 6 indicates very considerable contamination.

Single indices are indicators used to estimate single metal pollution by comparing the metal levels to its background levels. Alternatively, integrated indices, mainly based on single indices, are used to measure more than one metal pollution. In the current study, the Nemerow Pollution index (P_N_) was used as an integrated index to evaluate the comprehensive pollution status in sediment^84^. P_N_ allows the assessment of the overall degree of pollution of sediments and includes the contents of all investigated metals^85^. It is measured according to Eq. 2.

2 $${{\rm{P}}}_{{\rm{N}}}=\sqrt{({\overline{{\rm{CF}}}}^{2}+{{{\rm{CF}}}_{{\rm{Max}}}}^{2})/2}$$

where, $\,\overline{{\rm{CF}}}$ is the average of contamination factors of investigated metals and CF_max_ is the maximum contamination factor for a metal in a sample. According to Nemerow pollution index, the sediment quality was classified into five bands: P_N_ < 0.7, safe domain; 0.7 ≤ P_N_ < 1.0, warning domain; 1.0 ≤ P_N_ < 2.0, slightly polluted domain; 2.0 ≤ P_N_ < 3.0, moderately polluted domain; and P_N_ > 3.0, and seriously polluted domain^86^.

## Results

### Comparison of sediment quality with USEPA guidelines

**Figure 2:**
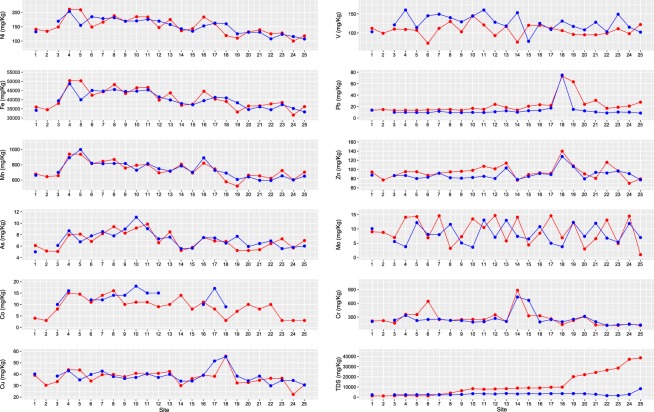
Spatiotemporal variations in concentrations of heavy metals in the Shatt Al-Arab River sediments during the dry season (red) and the wet season (blue).

**Figure 3:**
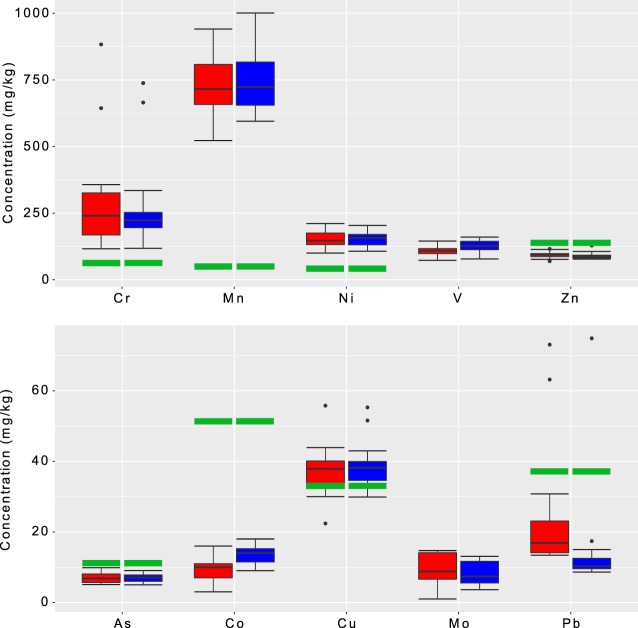
Box and Whisker for the heavy metals concentrations during the dry season (red box) and wet season (blue box). The horizontal black lines (inside the boxes) denote the medians of the concentrations. The bottom and top of the box show the first and third quartiles (Q1 and Q3). The whiskers are the lines inside the region defined by Q1-1.5(Q3-Q1) and Q3+1.5(Q3-Q1). The individual points with values outside these limits represent outliers. The horizontal green thick lines denote the USEPA thresholds. USEPA has no thresholds for V and Mo.

The sediment grain size (<63 µm fraction) in the current study ranges between 65%–87% of the total sediment sample size. The concentrations of heavy metals in the Shatt Al-Arab sediments showed considerable spatiotemporal variations (Fig. 2). A comparison of our data with sediment quality guidelines described by the United States Environmental Protection Agency^87^ revealed that concentrations of Ni, Mn, Cr, and Cu in all sites were higher than the USEPA threshold values (Fig. 3, Tables 1 and 2). The average concentration for Ni observed in the current study is 152 mg/kg, which is much higher than the USEPA maximum permissible value for Ni in river sediments (i.e., 22.7 mg/kg). Similarly, the average concentrations for Mn and Cr observed in this study are 735 and 264 mg/kg, respectively, which are much more than the USEPA guidelines for Mn and Cr (i.e., 30 and 43.4 mg/kg respectively). Cu average observed concentration is 37.9 mg/kg compared to 31.6 mg/kg of the USEPA threshold (Fig. 3, Tables 1 and 2). Site 18 and 19 have Pb concentrations of 74 and 39 mg/kg which are higher than the USEPA maximum permissible value for Pb in river sediments (i.e., 35.8 mg/kg) (Fig. 3, Tables 1 and 2).

**Table 1:** Heavy metals concentrations (mg/kg) in the Shatt Al-Arab River sediments in dry season (D) and wet season (W).

Site	Longi-tude	Lati-tude	WT		pH		TDS		Pb		Mo		Zn		Cu		Co		As		Ni		Mn		Fe		Cr		V	
			D	W	D	W	D	W	D	W	D	W	D	W	D	W	D	W	D	W	D	W	D	W	D	W	D	W	D	W
1	47°24′56.61′′E	31° 0′2.40′′N	29.1	10.8	8.7	8.1	1180	2320	13.6	13.7	9.0	10.1	94.3	87.6	39.1	40.2	4	ND	6.1	5.0	141	132	680	664	35955	34190	215	203	112	103
2	47°26′0.99′′E	31° 0′18.04′′N	28.4	10.9	8.8	NA	1170	NA	14.7	NA	8.8	NA	77.0	NA	30.4	NA	3.0	NA	5.2	NA	134	NA	646	NA	34505	NA	221	NA	99	NA
3	47°26′19.13′′E	31° 0′42.51′′N	28.2	10.9	8.3	8.0	1290	2300	13.4	9.9	7.0	5.6	86.4	86.6	33.6	38.4	8.0	10.0	5.1	6.1	149	169	658	701	37987	39412	168	231	110	121
4	47°26′31.00′′E	31° 0′17.06′′N	28.2	11.0	8.3	8.1	1350	2370	13.6	9.9	14.1	3.8	95.2	86.9	43.9	43.0	15.0	16.0	8.0	8.7	211	204	941	894	50377	48684	357	335	109	160
5	47°28′47.30′′E	30°59′10.31′′N	28.9	11.1	8.2	8.1	1250	2400	13.4	9.6	14.3	12.2	94.9	80.1	43.7	35.1	14.0	ND	8.1	6.8	209	155	939	1001	50301	39953	355	224	107	114
6	47°30′14.79′′E	30°54′12.06′′N	27.4	10.9	8.2	8.0	1210	2580	14.1	9.2	6.9	8.1	87.0	83.0	34.1	39.8	11.0	12.0	6.8	7.8	149	185	816	821	42418	45035	644	248	73	145
7	47°34′7.41′′E	30°49′9.71′′N	27.8	11.0	8.0	8.0	2610	2250	14.4	11.7	14.6	8.0	91.7	91.8	39.6	42.7	14.0	12.0	8.2	8.5	166	179	846	815	44291	44627	246	254	112	149
8	47°38′3.41′′E	30°46′3.37′′N	28.2	11.2	6.9	8.0	4030	2330	14.8	9.5	3.2	11.6	94.6	81.8	39.8	38	16.0	14.0	9.4	7.8	188	181	871	818	48240	45532	228	226	130	140
9	47°41′59.34′′E	30°44′57.35′′N	28.5	11.3	7.9	8.1	6340	2680	13.4	9.4	7.3	5.1	96.0	80.9	37.9	36.3	10.0	14.0	8.3	9.0	169	169	758	818	43397	44463	247	222	103	129
10	47°45′39.92′′E	30°39′54.85′′N	27.4	11.6	7.6	8.2	8350	3410	16.8	9.8	13.5	3.6	98.0	82.4	40.8	37.2	11.0	18.0	9.2	11.1	185	170	795	730	46475	44642	251	190	145	144
11	47°45′37.00′′E	30°37′51.68′′N	28.3	11.7	7.8	8.0	7770	3250	15.1	9.5	10.5	13.1	107.0	85.0	40.1	40.5	11.0	15.0	9.9	9.1	184	175	805	817	46685	45339	240	197	121	160
12	47°45′20.45′′E	30°34′16.89′′N	28.5	11.4	7.8	7.9	8060	3090	23.8	10.8	14.7	7.1	101.3	80.2	40.8	37.2	9.0	15.0	6.6	7.3	147	170	696	749	39759	41459	349	271	93	128
13	47°46′42.05′′E	30°34′41.32′′N	28.8	11.8	7.9	8.0	8330	3600	18.3	12.5	5.8	13.0	113.8	103.3	42.4	39.9	10.0	ND	8.5	7.6	175	158	716	719	43700	39827	203	206	117	118
14	47°47′57.07′′E	30°33′34.92′′N	30.8	11.9	7.3	8.1	8900	3140	13.6	10.1	14.1	7.4	76.6	78.0	30.0	34	14.0	ND	5.2	5.6	136	143	808	786	36930	37899	883	738	76	153
15	47°49′55.34′′E	30°32′25.36′′N	30.5	11.8	6.5	8.1	9070	3400	20.5	12.6	4.4	6.5	88.9	84.6	36.2	34.1	8.0	ND	5.8	5.6	143	134	694	703	37505	37201	326	665	120	78
16	47°51′3.54′′E	30°30′50.63′′N	29	11.7	7.2	8.0	9010	3190	23.1	13.1	8.5	10.8	92.5	91.1	39.1	39.2	11.0	10.0	7.5	7.5	184	153	822	892	44571	39449	328	193	120	125
17	47°51′31.16′′E	30°30′17.71′′N	29.1	11.6	6.7	7.9	9700	3440	21.9	17.4	14.6	5.0	91.5	88.9	38.2	51.6	8.0	17.0	6.9	7.4	160	163	747	728	40311	41237	261	246	112	109
18	47°56′32.68′′E	30°27′59.34′′N	28.5	11.4	7.5	8.1	9870	3480	73.1	74.9	6.9	3.8	140.0	128.3	55.8	55.3	3.0	9.0	6.8	6.5	119	160	579	693	39021	40918	136	196	106	131
19	48° 0′12.98′′E	30°27′43.76′′N	28.5	11.6	7.4	7.9	20160	3520	63.2	15.0	12.2	12.3	107.7	106.3	32.4	38.4	7.0	ND	5.2	7.7	110	125	522	609	33261	38292	225	252	96	117
20	48° 1′55.89′′E	30°27′25.27′′N	28.3	11.3	7.3	7.9	22120	3340	23.9	12.4	3.0	7.4	90.3	79.5	32.8	34.3	10.0	ND	5.2	5.9	132	131	665	643	36526	34612	311	316	95	108
21	48° 7′2.66′′E	30°25′47.04′′N	27.7	11.1	7.6	7.9	24280	2820	30.8	10.5	6.6	12.0	80.6	93.6	34.8	38.4	8.0	ND	5.4	6.5	139	131	656	597	36650	36081	127	197	95	128
22	48°15′38.18′′E	30°20′17.17′′N	27.5	10.9	7.8	8.1	26520	1490	16.9	8.7	13.1	6.8	115.5	92.2	36.5	29.9	10.0	ND	6.4	6.9	125	108	623	595	37676	34516	122	118	99	103
23	48°17′33.52′′E	30°18′47.26′′N	28.1	10.9	7.4	8.2	28600	1540	18.8	10.4	4.9	5.4	97.0	96.4	36.5	34.6	3.0	ND	7.3	5.6	128	123	726	656	38415	37305	118	131	111	149
24	48°29′22.34′′E	29°58′25.30′′N	29.2	10.7	7.8	8.2	37250	2760	20.8	10.1	14.5	11.9	69.7	90.9	22.4	34.6	3.0	ND	5.7	5.8	100	116	605	602	31590	35184	149	142	98	115
25	48°34′35.82′′E	29°56′39.65′′N	29.8	10.9	7.8	8.1	38640	8290	27.6	8.6	1.0	7.0	79.3	77.4	30.8	30.6	3.0	ND	7.0	6.0	118	107	704	652	36199	33307	116	127	122	102
Mean			28.6	11.3	7.7	8.0	11882	3041	22.1	13.7	9.3	8.2	94.7	89.0	37.3	38.5	9.0	13.5	6.9	7.1	152	152	733	738	40510	39965	273	255	107	126
SD			0.9	0.4	0.6	0.1	11417	1270	14.7	13.2	4.4	3.2	14.6	11.2	6.3	5.7	4.0	2.9	1.44	1.42	30.0	26	107	106	5249	4306	170	148	16	21

NA: stands for Not Available.

ND: stands for Not Detected.

**Table 2:** Comparison of the heavy metals concentrations in the sediments of the Shatt Al-Arab with historical values and international standards.

Heavy metal	Abaychi and Douabul 1985^33^	USEPA 2006^87^	Current study
Pb	16.1	35.8	18
Cr	107	43.4	264
Cu	33.9	31.6	37.9
Ni	103	22.7	152
Co	17	50	10.4
Zn	63	121	91.9
Mn	740	30	735
Fe	6800	20000	40243
As	—	9.8	7.1
Mo	2.6	—	8.8
V	182	—	117

### Spatial and temporal variations in river sediment quality

In general, heavy metals tend to have relatively low concentrations in sites 1, 2, and 3. Sites 1 and 2 represent the Euphrates River, whereas site 3 lies on the Tigris River (Figs. 1 and 2). The Shatt Al-Arab River course which starts from site 4 at the confluence of Euphrates and Tigris rivers, shows higher heavy metals concentrations compared to these two rivers (Fig. 2). Sites 4 and 5 exhibited high concentrations of Fe, Ni, Mn, As, Co, and Mo. Likewise, site 18 showed high Cu, Pb, and Zn concentrations. Along the river waterway, however, we observed a tendency of a gradual decrease in the levels of some metals towards the mouth of the river as the downstream stations have relatively low concentrations in terms of Fe, Ni, Mn, As, Co, and somewhat Cr (Fig. 2).

Our first approach to establishing the parameters associated with temporal variation was using the Spearman non-parametric correlation coefficient (Spearman’s R). To perform the Spearman R evaluation, each season was transformed to a numerical value in the data file (dry season = 1 and wet season = 2). This numerical variable was then correlated (pair by pair) with all the measured parameters. These bivariate results showed that among the 11 heavy metals investigated in the current study, only two metals exhibited significant correlations with the season. Pb and V displayed significant correlations with the season, i.e., Spearman’s R coefficient was -0.61 and 0.37 for these metals respectively. Other heavy metals showed no association with the season (Fig. 2). Water physical parameters (i.e., WT and TDS), however, exhibited significant correlations with the season, i.e., Spearman’s R coefficient was -0.87 and -0.48 respectively.

Water temperature exhibited higher values in the dry season compared to the wet season, with an average of 28.6 °C and 11.3 °C in these seasons respectively. WT was mainly constant along the river course during both seasons. TDS readings in the dry season have been displayed a gradual slight increase along the first 90 km of the river course (i.e., TDS increases from 1350 ppm to 9870 ppm throughout this upper reach). At a distance of 90 km (Sangar City), TDS exhibits a sharp increase (increases from 9870 ppm to 20160 ppm within 8 km) (Table 1; Fig. 2). In the wet season, nonetheless, TDS values were more consistent along the river waterway except for a gentle increase at site 24 (Table 1; Fig. 2). pH measurements revealed a steady trend in the wet season with an average of 8.0. On the other hand, pH in the dry season exhibited some anomalies at sites 8, 15, and 17, where these sites showed pH values smaller than other sites (Table 1).

### Statistical analysis

One of the most commonly used criteria for selecting the appropriate number of components in PCA is the Eigenvalue-one criterion^88^. In this approach, any component having an Eigenvalue above 1.00 should be retained and interpreted. The rationale for this criterion is that each observed variable contributes one unit of variance to the total variance in the entire data set. A component that displays an Eigenvalue above 1.00 is thus accounting for a larger amount of variance than has been contributed by one variable. Therefore, such a component is contributing to a meaningful amount of variance, and is worthy to be retained and interpreted^89–91^. In the present study, the components were considered as principal components when their Eigenvalues were higher than 1.00, and hence four principal components were selected. Equal numbers of varifactors (VFs) were extracted through the FA performed on the principal components. ^72^ classified the factor loadings as strong, moderate, and weak, corresponding to absolute loading values of > 0.75, 0.75-0.50, and 0.50-0.30, respectively. Among the four VFs obtained in the current study, VF1, with an Eigenvalue of 6.13 explaining 43.8% of the total variance, has a strong positive loading on Co, As, Ni, Mn, and Fe; and moderate loading on Cu, and V (Table 3). VF2 with an Eigenvalue of 1.98 explained 14.2% of the total variance, and showed strong moderate loading for WT, pH, and TDS. VF3 with an Eigenvalue of 1.17 explained 8.3% of the total variance, and exhibited strong loading for Zn and moderate loading for Pb. VF4 with an Eigenvalue of 1.1 explained 7.8% of the total variance, and displayed strong loading for Cr and moderate loading for Mo. The FA revealed that all investigated elements were well represented by the four VFs that explained a cumulative total variance of 74.08% (Table 3).

**Table 3:** Loadings of experimental variables (14) on significant principal components for the surface sediments samples of the Shatt Al-Arab River.

Component Element	VF1	VF2	VF3	VF4
WT	0.25	**−0.74**	0.35	0.23
pH	0.33	**0.55**	0.19	0.17
TDS	−0.17	**−0.71**	0.07	−0.37
Pb	−0.12	−0.41	**0.72**	−0.08
Mo	0.24	0.19	0.37	**0.48**
Zn	0.33	0.09	**0.84**	0.18
Cu	**0.72**	0.24	0.39	0.18
Co	**0.85**	−0.14	0.10	0.13
As	**0.87**	0.16	0.16	−0.01
Ni	**0.91**	0.11	0.07	0.29
Mn	**0.81**	0.10	−0.12	0.30
Fe	**0.94**	0.11	0.13	0.23
Cr	0.20	0.05	0.03	**0.84**
V	**0.62**	0.44	0.23	−0.31
Eigenvalue	6.13	1.98	1.17	1.10
% of variance	43.78	14.15	8.32	7.83
Cumulative %	43.78	57.93	66.25	74.08

Extraction Method: Principal Component Analysis.

Rotation Method: Varimax with Kaiser Normalization.

### Pollution analysis

**Figure 4:**
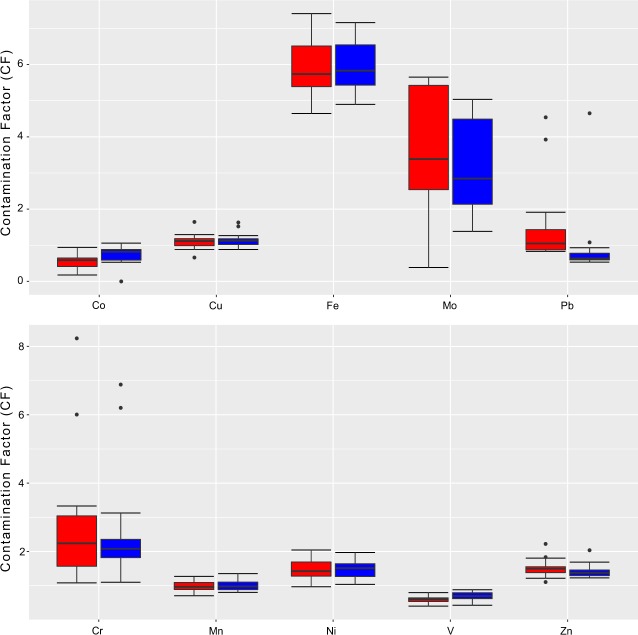
Box and Whisker showing CF of heavy metals during the dry season (red box) and wet season (blue box). The horizontal black lines (inside the boxes) denote the medians of the seasonal CFs. The bottom and top of the box show the first and third quartiles (Q1 and Q3). The whiskers are the lines inside the region defined by Q1-1.5(Q3-Q1) and Q3+1.5(Q3-Q1). The individual points with values outside these limits represent outliers.

**Figure 5:**
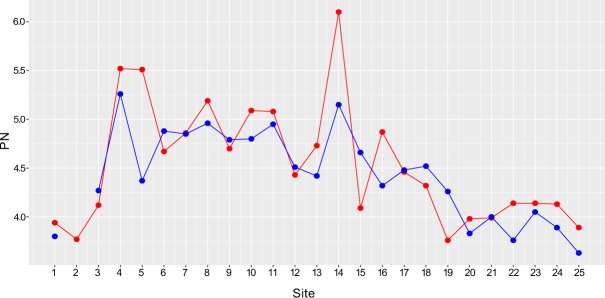
Seasonal variations of Nemerow Pollution index (P_N_) for metals at each sampling station during the dry season (red) and wet the season (blue).

In general, CF values of the measured metals revealed moderate to considerable contaminations. The CF values suggested that among all pollutants, Iron had the highest CF for the whole study area followed by Mo, Cr, Zn, Ni, Cu, Pb, Mn, Co, and V. Out of the 25 sampling sites, Fe showed very considerable contamination levels in 10 and 9 sites in the dry and wet seasons respectively, and considerable contamination levels in the other sites in both seasons (Table 4, Fig. 4). Mo displayed considerable contamination levels in 14 and 11 locations in the dry and wet seasons respectively, and moderate contamination levels in the other sites. Cr showed very considerable contamination levels in two sites in both dry and wet seasons, considerable contamination levels in five sites and one site in the dry and wet seasons respectively, and moderate contamination in the other sites. Zn, Ni, and Cu generally exhibited moderate contamination levels in all sites. During the dry season, Pb showed considerable contamination in two sites, moderate contamination levels in 12, and low contamination in the rest of the sites. On the other hand, Pb in the wet season exhibited considerable contamination in one site, moderate contamination in one site, and low contamination in the other sites. Co and V showed no contamination in terms of all investigated metals during the study period (Table 4, Fig. 4). The P_N_ values indicated serious pollution in the river sediments, as all sites displayed P_N_ values of greater than 3 in both dry and wet season (Table 4; Fig. 5).

**Table 4:** Contamination Factor (CF) and Nemerow Pollution index (P_N_) for Heavy metals in dry (D) and wet (W) seasons.

Site	Pb		Mo		Zn		Cu		Co		Ni		Mn		Fe		Cr		V		P_N_	
	D	W	D	W	D	W	D	W	D	W	D	W	D	W	D	W	D	W	D	W	D	W
1	0.8	0.9	3.5	3.9	1.5	1.4	1.2	1.2	0.2	ND	1.4	1.3	0.9	0.9	5.3	5.0	2.0	1.9	0.6	0.6	3.94	3.80
2	0.9	NA	3.4	NA	1.2	NA	0.9	NA	0.2	NA	1.3	NA	0.9	NA	5.1	NA	2.1	NA	0.5	NA	3.77	NA
3	0.8	0.6	2.7	2.2	1.4	1.4	1.0	1.1	0.5	0.6	1.4	1.6	0.9	0.9	5.6	5.8	1.6	2.2	0.6	0.7	4.12	4.27
4	0.8	0.6	5.4	1.5	1.5	1.4	1.3	1.3	0.9	0.9	2.0	2.0	1.3	1.2	7.4	7.2	3.3	3.1	0.6	0.9	5.52	5.26
5	0.8	0.6	5.5	4.7	1.5	1.3	1.3	1.0	0.9	0.0	2.0	1.5	1.3	1.4	7.4	5.9	3.3	2.1	0.6	0.6	5.51	4.37
6	0.9	0.6	2.7	3.1	1.4	1.3	1.0	1.2	0.6	0.7	1.4	1.8	1.1	1.1	6.2	6.6	6.0	2.3	0.4	0.8	4.67	4.88
7	0.9	0.7	5.6	3.1	1.5	1.5	1.2	1.3	0.8	0.7	1.6	1.7	1.1	1.1	6.5	6.6	2.3	2.4	0.6	0.8	4.86	4.85
8	0.9	0.6	1.2	4.5	1.5	1.3	1.2	1.1	0.9	0.8	1.8	1.8	1.2	1.1	7.1	6.7	2.1	2.1	0.7	0.8	5.19	4.96
9	0.8	0.6	2.8	2.0	1.5	1.3	1.1	1.1	0.6	0.8	1.6	1.6	1.0	1.1	6.4	6.5	2.3	2.1	0.6	0.7	4.7	4.79
10	1.0	0.6	5.2	1.4	1.6	1.3	1.2	1.1	0.6	1.1	1.8	1.6	1.1	1.0	6.8	6.6	2.3	1.8	0.8	0.8	5.09	4.80
11	0.9	0.6	4.0	5.0	1.7	1.3	1.2	1.2	0.6	0.9	1.8	1.7	1.1	1.1	6.9	6.7	2.2	1.8	0.7	0.9	5.08	4.95
12	1.5	0.7	5.7	2.7	1.6	1.3	1.2	1.1	0.5	0.9	1.4	1.6	0.9	1.0	5.8	6.1	3.3	2.5	0.5	0.7	4.43	4.51
13	1.1	0.8	2.2	5.0	1.8	1.6	1.3	1.2	0.6	ND	1.7	1.5	1.0	1.0	6.4	5.9	1.9	1.9	0.6	0.6	4.73	4.42
14	0.8	0.6	5.4	2.8	1.2	1.2	0.9	1.0	0.8	ND	1.3	1.4	1.1	1.1	5.4	5.6	8.2	6.9	0.4	0.8	6.1	5.15
15	1.3	0.8	1.7	2.5	1.4	1.3	1.1	1.0	0.5	ND	1.4	1.3	0.9	1.0	5.5	5.5	3.0	6.2	0.7	0.4	4.09	4.66
16	1.4	0.8	3.3	4.2	1.5	1.4	1.2	1.2	0.6	0.6	1.8	1.5	1.1	1.2	6.6	5.8	3.1	1.8	0.7	0.7	4.87	4.32
17	1.4	1.1	5.6	1.9	1.5	1.4	1.1	1.5	0.5	1.0	1.5	1.6	1.0	1.0	5.9	6.1	2.4	2.3	0.6	0.6	4.46	4.48
18	4.5	4.7	2.7	1.5	2.2	2.0	1.6	1.6	0.2	0.5	1.2	1.5	0.8	0.9	5.7	6.0	1.3	1.8	0.6	0.7	4.32	4.52
19	3.9	0.9	4.7	4.7	1.7	1.7	1.0	1.1	0.4	ND	1.1	1.2	0.7	0.8	4.9	5.6	2.1	2.4	0.5	0.6	3.76	4.26
20	1.5	0.8	1.2	2.8	1.4	1.3	1.0	1.0	0.6	ND	1.3	1.3	0.9	0.9	5.4	5.1	2.9	2.9	0.5	0.6	3.98	3.83
21	1.9	0.7	2.5	4.6	1.3	1.5	1.0	1.1	0.5	ND	1.3	1.3	0.9	0.8	5.4	5.3	1.2	1.8	0.5	0.7	3.99	4.00
22	1.0	0.5	5.0	2.6	1.8	1.5	1.1	0.9	0.6	ND	1.2	1.0	0.8	0.8	5.5	5.1	1.1	1.1	0.5	0.6	4.14	3.76
23	1.2	0.6	1.9	2.1	1.5	1.5	1.1	1.0	0.2	ND	1.2	1.2	1.0	0.9	5.6	5.5	1.1	1.2	0.6	0.8	4.14	4.05
24	1.3	0.6	5.6	4.6	1.1	1.4	0.7	1.0	0.2	ND	1.0	1.1	0.8	0.8	4.6	5.2	1.4	1.3	0.5	0.6	4.13	3.89
25	1.7	0.5	0.4	2.7	1.3	1.2	0.9	0.9	0.2	ND	1.1	1.0	1.0	0.9	5.3	4.9	1.1	1.2	0.7	0.6	3.89	3.63
Mean	1.4	0.9	3.6	3.2	1.5	1.4	1.1	1.1	0.5	0.7	1.5	1.5	1.0	1.0	6.0	5.9	2.5	2.4	0.6	0.7	4.54	4.43
SD	0.9	0.8	1.7	1.2	0.2	0.2	0.2	0.2	0.2	0.3	0.3	0.3	0.1	0.1	0.8	0.6	1.6	1.4	0.1	0.1	0.62	0.50

NA: Not Available.

ND: Not Detected.

## Discussion

### Comparison of sediment quality with USEPA guidelines

Sediment quality, a significant aspect of aquatic ecosystems, is mainly controlled by parent material^92^, land use^93,94^, climate^95^, and mixing with saline water due to tidal effect in lower reaches of coastal rivers^96,97^ as in the case of the Shatt Al-Arab River. The most notable land uses in the Shatt Al-Arab basin are industrial and commercial activities that accompanied the oil industry in the southern region of Iraq^98^. This is in addition to the residential and agricultural land uses in this region^99^. Land use types in a watershed have a critical impact on river sediment quality as land use is associated with natural processes and anthropogenic activities that manipulate the contaminant loads drained into river course^100^. While the growing oil industry in the Shatt Al-Arab basin has its consequences for the environment and communities, such industry has attracted more people to settle in the river basin. The population in the Shatt Al-Arab basin has doubled during the last two decades^1^. Such population growth, typically accompanied by residential wastes disposal, has added further load on the environment^101^. Anthropogenic effluents that are often discharged directly or flushed via runoff into the river are believed to be associated with producing toxic and hazardous substances into the river environment^102,103^. Moreover, the Mesopotamian Marshes draining into the Shatt Al-Arab that were once acting as a powerful filter for pollutants^14^ have tremendously degraded due to the extensive drainage and diversion of water supplies for agriculture, oil exploration, and military purposes in the 1990s^104^. International efforts have been made to restore the marshes since 2003, but restoration is patchy because of high soil and water salinities^105^. Furthermore, upstream dam projects now control the volume and timing of water coming into the marshes, and the total volume of incoming water has diminished^105–107^ found that the projected water yield reduction that causes lower discharge in the channel could affect both terrestrial and aquatic ecosystems. Thus, these natural purification systems (i.e., Mesopotamian Marshes) are still far from adequate restoration^15–17^. Therefore, the concentrations of heavy metals in the Shatt Al-Arab sediments (fraction <63 µm) in the present study showed considerably higher levels compared to historical values^33^ and USEPA guidelines^87^ (to be discussed in section pollution analysis) (Fig. 3, Table 2).

### Spatial variations in river sediment quality

Heavy metals are released into the environment by different anthropogenic activities^108^. The primary anthropogenic sources for heavy metals are industrial, agricultural, and residential activities^109^. Industrial actions, by which heavy metals are introduced into the environment, include fuel combustion, petroleum extraction, mining, smelting, metal finishing, and manufacturing waste disposal^110^. Agricultural activities such as applications of chemical fertilizers, animal manures, and pesticides containing heavy metals can significantly contribute to the metals polluting the environment^109^. Likewise, residential areas characterized by high population densities, excess energy consumption, and extended industrial and transportation activities are often characterized by large amounts of hazardous waste^111^. Basra governorate (the study area), the largest urban center in Southern Iraq, has various anthropogenic activities that most likely involved in heavy metals contamination^112,113^.

The spatial variations of the heavy metals concentrations in sediments along a river watercourse are a consequence of the different sources contributing to metal inputs^114^. Relatively low metals concentrations observed in the river’s tributaries (Euphrates and Tigris rivers), i.e., sites 1, 2, and 3 can be attributed to the fact that these tributaries are less contaminated compared to the Shatt Al-Arab. Right after the Euphrates and Tigris confluence at the Qurna City, heavy metals levels showed significant increases that extend to the middle parts of the river course (Fig. 2). The elevated metal concentrations in the sediments at the Qurna site may be caused by domestic wastewaters and industrial effluents discharged directly into the river^99^. Similar to our findings, ^99^ found high metals levels in the river sediments at the Qurna site, and ascribed that to the urban wastewater, oil deposits, corrosion of oil pipelines and floating bridges. In general, metals exhibited higher levels in the upstream parts, and showed a gradual decrease towards the river mouth. The high pollution levels in the upper and middle parts of the river detected in the current study can be attributed to the high population densities and industrial activities concentrated in these parts^115^. The present findings are in agreement with^116^, who stated that the primary sources of high metals levels in the upstream sites are sewage effluents, industrial wastes, oil spelling, and agricultural chemicals such as fertilizers and pesticides. Site 18 that corresponds to high metal levels (Fig. 2) receives pollution mostly from the nearby local metal workshops that withdraw sunken ships and boat wreck for disintegration into small metal scrap. This process requires cutting and welding, which, in turn, introduces such metals to the environment^117,118^.

The gradual decrease in concentrations towards the mouth of the river observed for Ni, Fe, Mn, As, Co, and somewhat Cu and Cr could refer to the common source of these metals that is more evident in the upstream parts compared to the downstream sites (Fig. 2). Another explanation for the relatively low pollution levels in the downstream sites is the intrusion of marine waters as well as the water turbulence and the erosion of the riverbed created by the higher current velocity in the southern parts of the river which can result in the reduction of toxic metals accumulation in the sediments^99^. The regressions of the gradual decrease in metals concentrations towards the mouth of the river observed in this study are similar to those reported by^83^, who reported a trend of a gradual reduction in heavy metals concentrations towards the Bay of Bengal. ^83^ attributed the relatively low heavy metals concentrations at the downstream stations to the high tidal activity throughout the year that these stations are experiencing. Similar findings were documented by^119^, who stated that the natural flushing action associated with tidal influences could result in a zone of low-level pollutants. Likewise^120^, found that the spatial distribution of heavy metals in coastal rivers can be controlled by the tidal hydrodynamic action.^120^ demonstrated that tidal flat has an individual self-purification capacity.

### Temporal variations in river sediment quality

Industrial and municipal wastewater effluents represent a continuous contamination source, whereas surface runoff constitutes a seasonal source, primarily influenced by climate conditions within river basin^36^. Seasonal variations in precipitation, surface runoff, and groundwater flow impose a substantial impact on river discharge and, consequently, on the concentrations of contaminants in river water^62,71^. It is widely reported that during rainy season, elevated river flows result in a dilution impact, and consequently, a decline in metals concentrations in river sediments. During dry season, however, river flow declines to cause an increase in the rate of sedimentation and ultimately the metals concentrations^39,83^ found relatively low metals concentrations in the Ganga River sediments in the wet season in comparison to the dry season and linked such a trend to the increased river flow during the wet season that results in dilution. Similar findings were documented by^38,121^ who found that the heavy metals exhibit higher concentrations in the dry season compared to the wet season and related this to the low flow rate during the dry season which promotes the precipitation and accumulation processes. In contrast to these studies that reported changes in heavy metals concentrations due to seasonal variations, our data showed no significant relationship between metals concentrations and seasonality.

^122^found lower sediment delivery during normal discharge conditions compared to extreme discharge conditions. Furthermore^123^, found that discharge could significantly control sediment load in the Loess Plateau as when discharge declines, whether due to climate change or anthropogenic activities, the sediment loads will subsequently decrease. The same scenario is most likely happening in the current study, i.e., the higher discharge during the wet season creates higher sediment yields from the adjacent areas considering the fact that these surrounding areas are remarkably polluted. Sediments, in turn, in untreated runoff from direct discharge storm water systems considerably contribute to heavy metals pollution in waterways^124,125^ and consequently sediments. ^126^reported high metals levels in the runoff and ascribed that to the metal breakthrough from the soil systems. Basra soil is known for high levels of heavy metals that exceed the international standard limits, and such high levels are often linked to the oil industry in the city^102^. The Shatt Al-Arab basin is characterized by intensive networks of intersecting creeks, sewage disposal inlets, and industrial waste ducts that represents a constant polluting source. During the wet season, runoff from a large area, including the Qurna and Basra cities, can generate huge amounts of metropolitan wastes stemming from residential and industrial sources. Such wastes are then transferred through creeks and drained into the river causing high pollutant levels in the river waterway. Similar findings were reported by^127,128^, who stated that heavy metal contamination in sediments is most likely to arise from the deposition of polluted sediments from the adjacent areas through land surface runoff. While the assumption that relatively high discharge in the wet season results in an additional dilution and consequently less pollution, such discharge increment will most likely promote the washout from heavily industrialized basins and thus introduces considerable amounts of pollutants to rivers^10^. In other words, the wet season is supposed to reduce the element concentrations in the river’s water and then sediment through dilution by increased discharge^129^. However, the additional dilution resulting from increased discharge may be offset by the flushing of uninterrupted deposition of waste in a basin with no proper sanitation infrastructure as the case of the Shatt Al-Arab basin^83^.

The hydrology of the Shatt Al-Arab basin is characterized by hot and dry summer and cold and rainy winter with a distinct tidal interplay phenomenon^11^. The water flow in the river is influenced by the tidal activities of the Arabian Gulf, which characterized by semi-diurnal patterns, with the tidal limit ranging from around one meter at the Basra City to three meters at the Fao City at the river mouth^130^. Furthermore, climate parameters in the Shatt Al-Arab basin notably vary throughout the year^20^. For example, the basin experiences relatively high rainfall and consequently higher discharge in the wet season. Alternatively, the basin has low to no rainfall outside of the wet season, and hence, the river discharge is relatively lower. The average river discharge in the dry season is 720 m^3^/s, and increases to 930 m^3^/s in the wet season^54^. Due to such discharge variations, the salt-wedge from the Arabian Gulf often extends up to 100 km upstream during the peak of the dry season and minimizes to less than 15 km in the wet season^98^. Thus, such seasonal variations can explain the trends of water temperature and TDS observed in the current study (Table 1; Fig. 2). Sharp TDS increase detected at station 18 in the dry season (Fig. 2) can be assigned to the high seawater intrusion in this season^98^. The average temperature in October and January (dry and wet seasons respectively) is 28 °C and 12 °C respectively^131^. Such change can explain the water temperature variations in our study since the water temperature of a natural stream is controlled by air temperature^132^. pH relatively lower levels at sites 8, 15, and 17 in the dry season can be ascribed to the anthropogenic activities at these sites. Industrial activities, especially power manufacturing from fossil fuels, can result in acidification of freshwater systems^133^. pH variations in the Shatt Al-Arab River were documented by^134^, and their anomalies (i.e., relatively low levels) were ascribed to anthropogenic activities in the river basin.

### Statistical analysis

Multivariate statistical methods such as principal component analysis and factor analysis are generally employed for pattern recognition, classification, and data dimensionality reduction^72,135–137^. In the current study, PCA and FA conducted on the normalized data sets (14 variables) have extracted four varifactors based on Eigenvalues. VF1 explained more than 40% of the total variance was dominated by Cu, Co, As, Ni, Mn, Fe, and V. These elements are most likely related to anthropogenic sources. In general, these metals showed relatively low concentrations at Tigris and Euphrates rivers, a sharp increase at the very origin of the Shatt Al-Arab, and then a gradual decrease in their levels from the middle parts of the river to the sea (Fig. 2). The elevated metals trend starting from site 4 to sites 18 and 19 is linked to heavily urbanized/industrialized areas surrounding the river course in this stretch. These metropolitan areas contribute to different sources of pollution, such as the discharging of industrial effluents, agricultural return flow, and untreated urban effluents^99^. Cu, As, and Ni can originate from fuel combustion and industrial emissions^109^. Anthropogenic sources of cobalt include fuel combustion, special steels, and metal mining and processing^138^. Mn can result from anthropogenic sources such as refining, smelting, fertilizer use, sewage sludge, and atmospheric deposition from fossil fuel combustion and waste incineration, which are common in the study area^139^. Iron in stream sediment often shows a very strong correlation with vanadium^140^ and that can explain their coexistence in the same group in the current study, and both can originate from industrial effluents^111^. VF2 represents the influence of water physical properties (i.e., WT, TDS, and pH) as these parameters were reported to be correlated to each other^141,142^. VF3 elements (i.e., Zn and Pb) are derived from anthropogenic effluents. Both metals show high levels in sites 18 and 19; sites that correspond to an industrial area containing metal welding workshops. The process of welding produces such metals to the environment^117,118^. The fourth varifactor VF4 is loaded on Mo and Cr, and can be attributed to agricultural sourcing. The primary sources of Mo in the environment are from the use of Mo fertilizers in agriculture^109^. Fertilizers may also contain several hundred to thousand ppm of Chromium^109^.

### Pollution analysis

Contamination factor analysis showed moderate to considerable Mo contamination in the study area. Mo anthropogenic contamination may occur from fertilizers and sewage sludge^138^. Large agricultural areas located on the banks of the river can explain the high Mo contamination levels reported in this study. High molybdenum concentrations in the environment, whether from natural sources or through pollution, has poisoning effect as excessive Mo levels can lead to bone deformation and disruption of metabolic processes in human and animals^143^. Cr also displayed high CF values ranging from moderate to considerable pollution. Highly toxic, causing liver and kidney damage and acting as a carcinogen, chromium can be introduced to the rivers by fertilizers^144^, and that can explain the high Cr levels in the river reaches that pass through agricultural lands (i.e., sites 4, 5, 6, 14, and 15). Zn and Pb high levels in specific sites (i.e., sites 18 and 19) are corresponding to an industrial area containing metal welding workshops which explain their relatively higher pollution levels in these sites^117,118^. Nickel high pollution levels in the upper reaches of the river can be attributed to sewage sludge and fuel combustion. The latter process has been identified to be the primary source of Ni in the environment, since petroleum contains considerable amounts of Ni^20^. Ni findings in this study are compatible with^112^ who found relatively high Ni pollution levels in Basra sediments and^103^ who also reported high Ni levels in northern parts of Basra. High Ni concentrations are both toxic, resulting in dermatitis and gastric irritation, as well as carcinogenic diseases^145^. From the Contamination Factor (CF) and Nemerow pollution index (P_N_) values, it was evident that the upper and middle parts of the river (i.e., sites 4 to 18) are relatively highly polluted sites (Table 4; Fig. 5). The crude oil production in Basra grew by four million barrels per day, rising from 0.5 million barrels per day in 1995 to almost 4.5 million barrels per day in 2017^146^. Such an oil industry expansion accompanied by extensive development in the northern parts of the Shatt Al-Arab drainage basin can contribute to the increasing metal pollution. Downstream reaches of the river are the least industrialized/populated areas, and thus receive relatively fewer pollution inputs which was confirmed by their lower CF and P_N_ values (Table 4; Fig. 5). Additionally, these sites most likely take advantage of self-purification due to tidal activities.

## Conclusions

Substantial amounts of anthropogenic pollutants were reported in the Shatt Al-Arab surface sediments in the current study. Concentrations of metals investigated such as Cr, Cu, Ni, Mn, and Fe were higher than the United States Environmental Protection Agency (USEPA) standards. Moreover, concentrations of Pb, Cr, Cu, Ni, Zn, Fe, and Mo in the current study were higher than the local background levels (i.e., the year 1985), indicating that the river is experiencing considerable pollution. In contrast to the previous studies that reported changes in heavy metals concentrations in sediments due to seasonal variations, our data showed no significant relationships between metals concentrations and seasonality. While the assumption that relatively high discharge in the wet season results in an additional dilution and consequently less pollution, such additional dilution may be offset by the wet season flushing of uninterrupted deposition of waste in a basin with no proper sanitation infrastructure. Statistical analysis revealed that most metals are of anthropogenic sourcing. Metals such as Cu, Co, As, Ni, Mn, Fe, V, Zn, and Pb are mainly related to industrial and residential activities such as fuel combustion, metal mining, processing, refining, smelting, and sewage sludge disposal. On the other hand, Molybdenum and Chromium are most likely of agricultural origin (i.e., agricultural fertilizers). Furthermore, pollution analysis displayed high sediment pollution in terms of several metals Fe, Mo, Cr, Zn, and Ni. The pollution, however, was not evenly distributed along the river course as the highest levels of heavy metals were reported at the upper and middle reaches of the river, and were attributed to the higher industrial and urban development in these parts. Downstream reaches of the river, on the other hand, are the least industrialized/populated areas, and thus receive relatively fewer pollution inputs. Furthermore, downstream sites are most likely to take advantage of self-purification and assimilative capacity of a river with powerful tidal activities.
